# Relationship between Leadership and Emotional Intelligence in Teachers in Universities and Other Educational Centres: A Structural Equation Model

**DOI:** 10.3390/ijerph17010293

**Published:** 2019-12-31

**Authors:** Félix Zurita-Ortega, Eva María Olmedo-Moreno, Ramón Chacón-Cuberos, Jorge Expósito López, Asunción Martínez-Martínez

**Affiliations:** 1 Department of Didactics of Musical, Plastic and Corporal Expression, University of Granada, 18071 Granada, Spain; felixzo@ugr.es; 2 Department of Research Methods and Diagnosis in Education, University of Granada, 18071 Granada, Spain; emolmedo@ugr.es (E.M.O.-M.); jorgeel@ugr.es (J.E.L.); asuncionmm@ugr.es (A.M.-M.)

**Keywords:** leadership, emotional intelligence, teaching, university

## Abstract

This study uses an explanatory model of the dimensions of leadership and emotional intelligence according to the methods used in particular teaching environments (universities and other educational institutions). The effect of different kinds of leadership on emotional intelligence dimensions is also established using an explanatory model. A total of 954 teachers participated in this cross-sectional study, teaching in 137 different schools/universities. The instruments used for the data collection were the Multifactor Leadership Questionnaire (MLQ-5) and the Trait Meta Mood Scale (TMMS-24). Data analysis was performed with the software IBM AMOS 23.0. (International Business Machines Corporation, Armonk, NY, USA) using multi-group analysis and structural equations. Results showed that the structural equation model had a good fit. Transformational leadership depends mainly on intellectual stimulation in university teachers, whereas intrinsic motivation is more relevant at the lower educational levels. In relation to transactional leadership, contingency reward has a greater regression weight in non-university education, whereas passive leadership is governed more by passive exception in university teachers. There was a positive and direct relationship between levels of emotional intelligence and transformational leadership in non-university teachers, which reveals the need for effective understanding and management of both one’s own and students’ emotions in order to act effectively as a leader. Transactional leadership was negatively related to some emotional intelligence dimensions, given the relevance of obtaining power in this dimension.

## 1. Introduction

Teachers’ personal traits as well as their professional skills are significant in relation to bringing about behavioral changes in students [1]. Among the factors justifying the training of teachers in emotional ability in all types and at all stages of education, it can be highlighted the need to carry out teaching tasks with emotional intelligence, as well as the need to teach emotional skills to students. These ideas concur with studies on students and teachers in secondary education in several contexts [2,3].

In light of the scientific literature on this topic, above all focusing on emotional understanding and control skills, these factors are considered predictors of a better management of everyday life and are related to higher levels of well-being and psychological adjustment [4,5,6,7]. Other studies, such as those by Mearns and Cain [8], revealed that teachers with high expectations of being able to manage their negative emotions used more coping strategies. Additionally, Rodríguez-Corrales et al. [9] analyzed the direct effect of non-university teachers’ emotions on the assessment of students’ performance.

Notwithstanding the various viewpoints from which emotional intelligence and positive psychology have been studied, it can be seen that almost no studies address leadership as indicated by Sánchez et al. [10], despite the fact that psychological theories highlight the importance of practice, reasoning and management. Nevertheless, several authors have shown the importance of teachers’ emotional intelligence and their correct use in order to improve thinking and management capacity [11,12]. In fact, high levels of emotional well-being have been developed in other professions, such as nursing and odontology, in order to get high levels of leadership, which is really important in these professions [3,13].

A positive relationship was found between emotional intelligence and the different styles of educational leadership [1]. In fact, various studies have confirmed that those teachers who are emotionally intelligent can manage and direct in a better way the daily challenges presented by students and by the teaching-learning process more effectively and positively [14].

Recently, the term leadership has come to represent one of the most important and recurrent lines of research in studies on organizations in general and on educational institutions in particular [15,16,17,18]. Most works and studies analyzed confirm the positive effect that leadership has on many and varied aspects, notably educational level and teachers’ results [19,20,21].

The idea of implementing emotional intelligence as a tool to improve teachers’ leadership and well-being has been considered in almost all educational institutions worldwide. Specifically, Asrar-ul-Haq et al. [11] have carried out research on the way intelligence influences teachers’ ways of working in Pakistan, while Vinichenko et al. [22] analyze differences related to the development of work and suggest measures for optimizing teachers’ training. A study by Ozdemir and Kocak [23] determined that human behavior in any working environment is associated with leadership styles and establishes a more positive emotional charge in teachers with academic posts.

In this way, several researches determine the existence of different types of leadership [19,20,21,22,23]. Specifically, passive leadership, which is based on letting the student perform the tasks without help; democratic leadership, whereby the students take part in the decisions about the learning process in educational context, and transformational leadership, based on intellectual stimulation and the idealized influence. Thus, this study aims to analyse the relationships between these three types of leadership and the basic skills of emotional intelligence, including the perception, regulation, and use of emotions.

**Figure 1:**
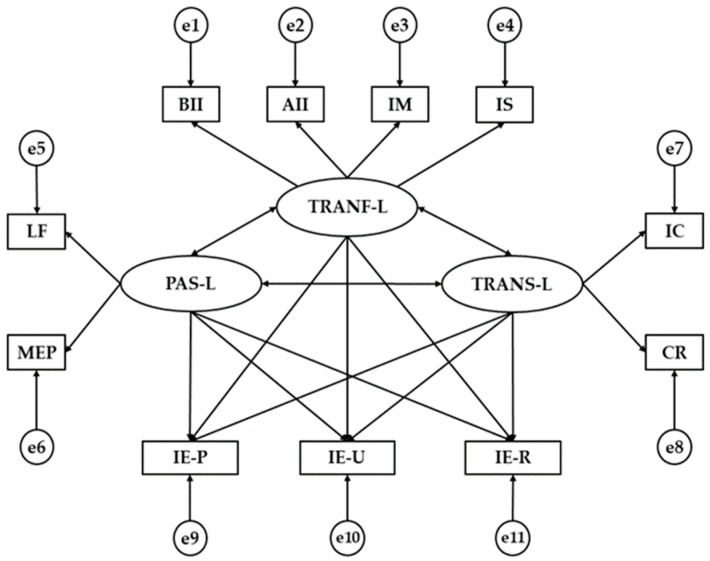
Theoretical model. Note1: TRANF-L, Transformational Leadership; TRANS-L, Transactional Leadership; PAS-L, Passive Leadership; BII, Behavioral Idealized Influence; AII, Attributed Idealized Influence; IM, Inspired Motivation; IS, Intellectual Stimulation; IC, Individualized Consideration; CR, Contingent Reward; MEP, Management-by-Exception Passive; LF, Laissez-Faire; IE-P, Emotional Intelligence Perception; IE-U, Emotional Intelligence Understanding; IE-R, Emotional Intelligence Regulation.

Bearing all this in mind, this study is based on the following theoretical assumption and hypothetical model (Figure 1) with the following factors: Factor 1: Transformational Leadership (TRANF-L); Factor 2: Transactional Leadership (TRANS-L); Factor 3: Passive Leadership (PAS-L); Factor 4: Behavioral Idealized Influence (BII); Factor 5: Attributed Idealized Influence (AII); Factor 6: Inspired Motivation (IM); Factor 7: Intellectual Stimulation (IS); Factor 8: Individualized Consideration (IC); Factor 9: Contingent Reward (CR); Factor 10: Management-by-exception: passive (MEP); Factor 11: Laissez-Faire (LF); Factor 12: Emotional Intelligence-Perception (IE-P); Factor 13: Emotional Intelligence-Understanding (IE-U); Factor 14: Emotional Intelligence-Regulation (IE-R).

The model that has been developed aims to ascertain the connection between the emotional intelligence dimensions (perception, understanding and regulation) of teachers and their different leadership styles, since various studies show a close relationship between them [24,25,26]. Additionally, the study aims to establish the connections between the three styles of leadership, since studies such as those by Griffioen and De Jong [27], Elrehail [28], or Hassan et al. [29] address how important, in terms of management, is the exchange of knowledge between different types of leadership and innovation in higher education institutions, and above all in teaching itself. Herein some of the errors detected having been attributed to poor-quality leadership.

It is also convenient to eliminate connections between dimensions in the TMSS-24, since each of the parameters (perception, understanding, and regulation) is an independent entity. In the same regard, and according to Marsh [30], it is convenient and necessary to eliminate the less significant connections, provided that the fit indices in the model are not affected, in order to obtain a synthesized and concise model (Figure 1).

In the proposed model, passive leadership, transformational leadership, and transactional leadership act as exogenous variables, whereas behavioral idealized influence, attributed idealized influence, inspired motivation, intellectual stimulation, individualized consideration, contingent reward, management-by-exception passive, laissez-faire, emotional intelligence perception, emotional intelligence understanding, and emotional intelligence regulation act as endogenous variables. Two-way arrows (covariances) connect exogenous variables, whereas one-way arrows reveal the effects (direct and indirect) between endogenous variables. Associations are established between prediction errors in the endogenous variables, which receive the effects of others and need errors in variables. Estimation of the parameters was carried out using the maximum likelihood estimation method (ML), which is coherent, non-biased and unaffected by the type of scale. Thus, this study has as its main objectives: (a) to analyze and determine the relationship between leadership and emotional intelligence in Spanish teachers; (b) to define and contrast an explanatory model of the dimensions in emotional intelligence according to leadership styles and type of teaching (in universities or otherwise), and (c) to analyze the effect of leadership styles on emotional intelligence dimensions using a multigroup explanatory model according to different educational levels.

## 2. Materials and Methods

### 2.1. Subjects and Design

This descriptive and cross-sectional research project was carried out on a sample of 954 teachers in Spain, both men and women (45.9%, 438 men and 54.1%, 516 women), from 137 schools, with different typologies and levels (Infant, Primary, Secondary, Training Courses, Universities and others). The sample was chosen by random sampling among teachers from all educational levels in Spain (N = 691,235), being 117,716 university teachers and 573,519 teachers at other levels. The sampling error was acceptable, specifically 0.03 for the overall sample (0.06 for university teachers and 0.04 for the rest). It should be noted that 65 questionnaires were invalidated because they were not properly completed.

### 2.2. Instruments

Three different types of instrument were used in this project. The first was a self-completion questionnaire where the teachers indicated their gender, their age, and their seniority or the educational level at which they taught (Infant, Primary, Secondary, Training Courses, University, and others, into which category came teachers of religious education, School Counselors, Special Needs, Adult Education teachers, etc.).

The second instrument used was the MLQ-5X by Bass & Avolio [31], named in its original version Multifactor Leadership Questionnaire, which is considered one of the most effective instruments for measuring leadership [32]. This questionnaire is made up of 45 items, measured using a Likert scale with five options, whereby 0 is “Totally disagree” and 4 is “Totally agree”. The first 36 items identify leadership behavior in the leaders (Transformational, Transactional and Laissez-Faire), and items 37 to 45 are in response to those leadership results. The instrument had a Cronbach’s Alpha of α = 0.879.

The third instrument is the TMMS-24, which is based on the Trait Meta Mood Scale (TMMS-24) proposed by Salovey, Mayer, Goldman, Turvey, & Palfai [33]. The original scale assesses emotional states through 24 items, structured in three dimensions (perception, understanding and regulation) with 8 items each, and answered using a Likert scale with five options. In Spain, it has been used by Cazalla-Luna and Molero [34]. In this study, the Cronbach’s Alpha was α = 0.864.

### 2.3. Procedure

Firstly, all the teachers in Spain were counted. Then, the Faculty of Education of the University of Granada (Spain) composed an explanatory letter inviting people to collaborate. This document explained the nature and objectives of the study and requested the consent of those willing to participate. Once an affirmative reply had been received, the questionnaire was sent by email so that participants could answer frankly; in some cases, on request, the questionnaire was mailed in paper form. A total number of 1019 teachers took part in this project, 65 questionnaires being invalidated because they were not correctly completed. The instruments were applied from January to March 2018. Anonymity and confidentiality of data was ensured. Data were collected and its quality was confirmed, whilst ensuring throughout that the process conformed to the ethical principles for research defined in the Declaration of Helsinki in 1975 and later updated in Brazil in 2013. 

### 2.4. Statistical Analysis

For data analysis, the statistical software IBM SPSS 24.0. (International Business Machines Corporation, Armonk, NY, USA) was used in order to establish the values of the basic descriptors (means and frequencies). For the analysis of relations and effects existing between the constructs of the structural model, program IBM AMOS 23.0. (International Business Machines Corporation, Armonk, NY, USA) was used, carrying out a multigroup analysis. A model of route analysis was created with the following observable variables: transformational leadership (LTRANSF); transactional leadership (LTRANSA); passive Leadership (LPASIVO); Behavioral Idealized Influence (IIC); attributed idealized influence (IIA); inspired motivation (MI); intellectual stimulation (EI); individualized consideration (CI); contingent reward (RC); management-by-exception: passive (DEP); laissez-faire (LF); emotional intelligence perception (PERIE); emotional intelligence understanding (COMIE); emotional intelligence regulation (REGIE).

Model fit was checked in order to verify compatibility and the empirical information obtained. Fit reliability was assessed using Marsh’s goodness-of-fit indices, which indicate that in Chi-square, non-significant values associated with p denote a good model fit. The comparative fit index (CFI) will be acceptable if higher than 0.90, and excellent if higher than 0.95. The normed fit index (NFI) must be higher than 0.90; the Incremental Fit Index (IFI) will be acceptable if higher than 0.90 and excellent if higher than 0.95. Finally, the root mean square error of approximation (RMSEA) will be excellent if lower than 0.05 and acceptable if lower than 0.08.

## 3. Results

The descriptive study of data relating to the 954 participants established that values were similar by gender. The greater proportion of non-university teachers is also significant, most such participants having worked as a teacher for less than 20 years. As regards the distribution of teachers according to education type, there were more men (35.6%) than women in higher education, the proportions being reversed in all other types of education. Regarding the length of service, there is a greater presence of senior teachers in higher education, as can be seen in the following table (Table 1).

The proposed structural equation model obtained a good fit in all assessment indices. The Chi-square had a significant value of p (χ^2^ = 273.540; gl = 70; *p* < 0.001). Nevertheless, this index cannot be interpreted in a standardized way, added to which a problem is posed by its sensitivity to sample size [30]. Therefore, other standardized fit indices less sensitive to sample size were used. The comparative fit index (CFI) had a value of 0.918, which was acceptable. The normed fit index (NFI) had a value of 0.903 and the incremental fit index (IFI) a value of 0.905, both also acceptable. The root mean square error of approximation (RMSEA) had an acceptable value of 0.078.

**Figure 2:**
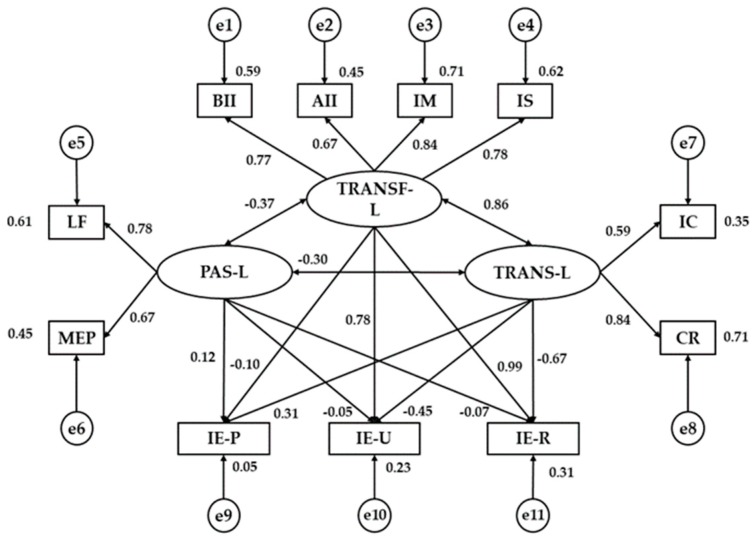
Structural equation model for inferior teachings. Note1: TRANF-L, Transformational Leadership; TRANS-L, Transactional Leadership; PAS-L, Passive Leadership; BII, Behavioral Idealized Influence; AII, Attributed Idealized Influence; IM, Inspired Motivation; IS, Intellectual Stimulation; IC, Individualized Consideration; CR, Contingent Reward; MEP, Management-by-Exception Passive; LF, Laissez-Faire; IE-P, Emotional Intelligence Perception; IE-U, Emotional Intelligence Understanding; IE-R, Emotional Intelligence Regulation.

Figure 2 and Table 2 show estimated values of the parameters in the model for teachers in preschool and primary levels. These must be of a suitable magnitude and the effects must be significantly different from zero. No improper estimations such as negative variances should be found. Statistically significant relations are found at the *p* < 0.005 level in all associations between Transformational Leadership and its indicators, all of these being positive and direct and showing a stronger correlation for Inspired Motivation (r = 0.844) and Intellectual Stimulation (r = 0.784). Likewise, the same tendency is found in Passive Leadership (*p* < 0.005), with a stronger correlation in Laissez-faire (r = 0.780) and Transactional Leadership (*p* < 0.005), Contingent Reward being the variable with the highest positive correlation in relation to its dimension (r = 0.842).

Next, relations between the different leadership dimensions were analyzed. A positive and direct relationship between Transformational and Transactional Leadership is found (r = 0.863), the level being significant at *p* < 0.005. In contrast, Passive Leadership is inversely related to Transformational Leadership (r = −0.375) and to Transactional Leadership (r = −0.298), both being significant at a level of *p* < 0.01.

Checking associations between Leadership and Emotional Intelligence, statistically significant differences are revealed between Transformational Leadership and Emotional Intelligence Regulation (r = 0.986), and between Transformational Leadership and Emotional Intelligence Understanding (r = 0.785), both positive and direct. The same tendency is displayed in the relation between Transformational Leadership and Emotional Intelligence Perception (r = 0.311). Likewise, statistically significant differences are found in level *p* < 0.01 between Transactional Leadership and Emotional Intelligence Regulation (r = −0.673), and between Transactional Leadership and Emotional Intelligence Understanding (r = −0.447), both negative and inverse. Finally, no association was found between Passive Leadership and the three dimensions of Emotional Intelligence (Perception, Understanding, and Regulation).

**Figure 3:**
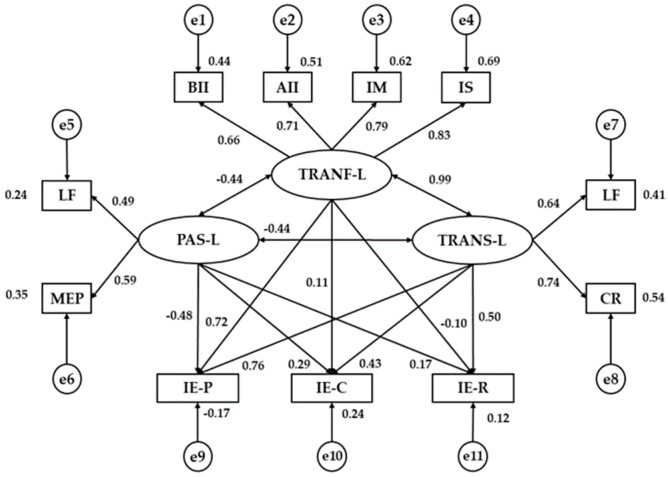
Structural equation model for university teaching. Note1: TRANF-L, Transformational Leadership; TRANS-L, Transactional Leadership; PAS-L, Passive Leadership; BII, Behavioral Idealized Influence; AII, Attributed Idealized Influence; IM, Inspired Motivation; IS, Intellectual Stimulation; IC, Individualized Consideration; CR, Contingent Reward; MEP, Management-by-Exception Passive; LF, Laissez-Faire; IE-P, Emotional Intelligence Perception; IE-U, Emotional Intelligence Understanding; IE-R, Emotional Intelligence Regulation.

Figure 3 and Table 3 show estimated values of the parameters in the model for university teachers. These must be of an adequate magnitude and the effects must be significantly different from zero. No improper estimations such as negative variances should be found. There are statistically significant relations at the *p* < 0.005 level for all the associations between Transformational Leadership and the indicators in this dimension, all of them being positive and direct, the factors having the strongest correlation being Inspired Motivation (r = 0.787) and Intellectual Stimulation (r = 0.829). Likewise, the same tendency is displayed for Passive Leadership (*p* < 0.005) with a stronger correlation in Management-by-exception Passive (r = 0.589), and in Transactional Leadership (*p* < 0.005), with Contingent Reward being the variable with the highest positive correlation with its dimension (r = 0.738).

Next, the relations between the different leadership dimensions are analyzed. In the first place, a positive and direct relation is revealed between Transformational Leadership and Transactional Leadership (r = 0.997), being significant at level *p* < 0.005. In contrast, Passive Leadership is inversely related to Transformational Leadership (r = −0.441) and Transactional Leadership (r = −0.443), both being significant at level *p* < 0.01.

Analyzing the associations between leadership and Emotional Intelligence, the only statistically significant difference found was between Passive Leadership and Emotional Intelligence Understanding, being positive and direct (r = 0.292; *p* < 0.05).

## 4. Discussion

This study, conducted on a sample of 954 teachers working at different educational levels, focused on two aspects: on the one hand leadership, which has been a topic of discussion since time immemorial, its particular interest to thinkers and scientists being that it is the central axis of administration and organizational behavior; and on the other hand emotional intelligence, which is an essential element in positive psychology. In order to discover the cause and effect relationships of the theoretical assumption suggested, two groups were created: university teachers and teachers working at other educational levels (non-university teachers).

From the findings obtained herein, it can be deduced that the most influential indicator of transformational leadership in non-university teachers is intrinsic motivation, attributed idealized influence exerting the least influence. This could be related to the vocational component inherent in education at basic levels (Infant and Primary), in line with the studies by Evers et al. [35] and Andersson and Kopssen [36], who stress the importance of teacher’s behavior being autonomous and of this being linked to professionalism and vocation. In the same way, the general principle of attributed idealized influence relies on the teacher being a respected and admired leader who enjoys the confidence of those who have conferred on him or her the role of leader. The fact that this applies much less to non-university education explains why in that sector attributed idealized influence is valued least in terms of transformational leadership.

In the case of university teachers, the most relevant indicator of transformational leadership is intellectual stimulation. This may be due to teaching strategies that aim to generate problems and get students to solve them in the context of higher education [37]. This type of work evolves rapidly, and teachers need to keep abreast of changes taking place and progress being made, constantly updating their teaching and intellectual stimulation. Thus, university teachers feel the need for professional self-development, an integral component of which is the combination of intellectual and affective factors, intelligence, and will [36,38]. At the other end of the scale, the weakest correlation was found in behavioral idealized influence.

With regard to passive leadership, it should be noted that the Laissez-faire dimension was the most relevant for non-university teachers, since at basic levels it is common to use methodologies based on the discovery and self-management of resources and tasks, with the aim of acquiring certain skills [39]. In addition, at the various educational stages, each teacher is freer to apply different methodologies, motivated by giving students greater decision-making power so that they can develop positive thinking, despite leadership being associated with lower performance when the leader is not there [40]. Conversely, in the case of university teachers, there is a stronger correlation with passive management-by-exception, which is characterized by the leader’s intervention when a problem is identified or when help is requested by the group [16].

In relation to transactional leadership, contingent reward is the most influential factor in both types of teacher, although the correlation is stronger for teachers at the lower educational stages. This is common, given that contingent reward is based on the positive reinforcement of appropriate behavior, which is a widely used strategy for controlling contingency in infant and primary education [41]. By contrast, in higher education, academic behaviors are usually well established. Therefore, the use of contingency control techniques is less common, instead, motivational strategies with different levels of self-determination are used [42]. Equally, university teachers tend not to discuss their working practices or readily adopt innovations, which could be another reason for the low correlation, as suggested by Koeslag-Kreunen, Van der Klink, Van de Bossche, & Gijselaers [43].

Analysis of the links among leadership dimensions showed a positive and direct relationship between transactional and transformational dimensions, which was stronger in university teachers. Transformational leadership is characterized by a leader’s desire to bring about change, and in order to do so, he or she needs to be granted authority by the group. Similarly, transactional leadership is characterized by increasing empowerment of the leader as positive benefits for the group are achieved, evidencing the relationship between the two types of leadership [32]. Some university teachers are more charismatic and others more liberal, but passive leaders are scarce since the teaching profession does not allow this attitude, which substantiates these findings [44].

In the case of university teachers, there is little connection between emotional intelligence and leadership, the only positive relationship being that between passive leadership and emotional understanding, which implies that these teachers have less need to understand emotions in order to act as leaders. In the case of non-university teachers, this relationship is not statistically significant, contrary to the findings of Majeed et al. [24], which pointed to there being important and positive aspects to emotional intelligence on the organizational side of higher education, in contrast to secondary education. In fact, Tsvetkova [38] posited that university teachers’ intelligence contains self-knowledge components based on self-respect, sociability, emotional regulation, and self-acceptance [20]. 

It is notable that in non-university teachers there are various connections between leadership and emotional intelligence that are not found in university teachers. As suggested by Halitsan et al. [45], as part of their training, prospective teachers are being taught pedagogical facilitation (empathy, emotional intelligence, pedagogical communication, motivation in teaching, etc.), suitable for use in the educational context of modern schools, which can help them become effective leaders [43,46]. 

Finally, transformational leadership was positively related to emotional understanding and emotional regulation, having a strong positive and direct association that was not found in university teachers, thus confirming the conclusions of Majeed et al. [24]. Likewise, both transactional leadership and transformational leadership were positively related to emotional perception and emotional understanding, respectively, again demonstrating that those associations are not found in university teachers. In contrast, transactional leadership was negatively related to emotional understanding and regulation in non-university teachers, which seems logical in that the basis of this type of leadership is greater power for the leader [32]. 

The data obtained from this study show the importance of conducting studies in this sphere, since, from the point of view of positive psychology and leadership, it is interesting to discover the influence of emotional intelligence on teachers’ ability to act as leaders, not only in higher education, but at all educational levels. One of the limitations of the study is that the multigroup analysis could only be performed on two groups, so a breakdown of the associations between variables for each of the different educational levels would give the study greater richness. Another limitation is the representativeness of the various groups, there having been substantial participation by infant, primary, secondary and university teachers, which was not the case in other categories (training courses or education for adults, among others).

## 5. Conclusions

The main conclusions of this study are:

- Transformational leadership depends mainly on intellectual stimulation in university teachers, whereas intrinsic motivation is more relevant at the lower educational levels. In relation to transactional leadership, contingency reward has a greater regression weight in non-university education, whereas passive leadership is governed more by passive exception in university teachers.
- There is a positive and direct relationship between transactional leadership and transformational leadership, which is stronger in university teachers. Additionally, both are inversely related to passive leadership at all levels.
- There is a positive and direct relationship between levels of emotional intelligence and transformational leadership in non-university teachers, which reveals the need for effective understanding and management of both one’s own and students’ emotions in order to act effectively as a leader. Transactional leadership was negatively related to some emotional intelligence dimensions, given the relevance of obtaining power in this dimension.

## Figures and Tables

**Table 1:** Descriptive study.

	Teaching	Higher Teaching	Other Teachings	Total
**Male**	Count	156	282	438
	% Gender	35.6%	64.4%	100.0%
	% Teaching	56.5%	41.6%	45.9%
**Female**	Count	120	396	516
	% Gender	23.3%	76.7%	100.0%
	% Teaching	43.5%	58.4%	54.1%
**Experience (<5 ages)**	Count	60	213	273
	% Experience	22.0%	78.0%	100.0%
	% Teaching	21.7%	31.4%	28.6%
**Experience (5–10 ages)**	Count	24	149	173
	% Experience	13.9%	86.1%	100.0%
	% Teaching	8.7%	22.0%	18.1%
**Experience (10–15 ages)**	Count	78	184	262
	% Experience	29.8%	70.2%	100.0%
	% Teaching	28.3%	27.1%	27.5%
**Experience (15–20 ages)**	Count	66	90	156
	% Experience	42.3%	57.7%	100.0%
	% Teaching	23.9%	13.3%	16.4%
**Experience (>20 ages)**	Count	48	42	90
	% Experience	53.3%	46.7%	100.0%
	% Teaching	17.4%	6.2%	9.4%
**Total**	Count	276	678	954
	% Experience	28.9%	71.1%	100.0%
	% Teaching	100.0%	100.0%	100.0%

**Table 2:** Regression weights and standardized regression weights for inferior teaching.

Relationships between Variables			R.W				S.R.W
			Estimate	E.E.	C.R.	*p*	EST
BII	←	TRANF-L	1.000	-	-	***	0.769
AII	←	TRANF-L	1.147	0.093	12.381	***	0.669
IM	←	TRANF-L	1.271	0.079	16.046	***	0.844
IS	←	TRANF-L	1.015	0.069	14.815	***	0.784
MEP	←	PAS-L	1.000	-	-	***	0.674
LF	←	PAS-L	1.046	0.198	5.284	***	0.780
IE-P	←	TRANF-L	−0.140	0.228	−0.616	0.538	−0.099
IE-R	←	TRANF-L	1.331	0.281	4.732	***	0.986
CR	←	TRANS-L	1.648	0.164	10.072	***	0.842
IC	←	TRANS-L	1.000	-	-	***	0.588
IE-P	←	PAS-L	0.135	0.081	1.657	0.097	0.120
IE-R	←	TRANS-L	−1.215	0.376	−3.232	***	−0.673
IE-U	←	TRANF-L	1.160	0.256	4.524	***	0.785
IE-P	←	TRANS-L	0.587	0.307	1.912	**	0.311
IE-U	←	TRANS-L	−0.884	0.340	−2.601	**	−0.447
IE-R	←	PAS-L	−0.076	0.078	−0.973	0.331	−0.071
IE-U	←	PAS-L	−0.062	0.083	−0.746	0.455	−0.052
TRANF-L	↔	PAS-L	−0.077	0.018	−4.292	***	−0.375
TRANS-L	↔	TRANF-L	0.106	0.014	7.568	***	0.863
TRANS-L	↔	PAS-L	−0.046	0.014	−3.381	***	−0.298

Note1: TRANF-L, Transformational Leadership; TRANS-L, Transactional Leadership; PAS-L, Passive Leadership; BII, Behavioral Idealized Influence; AII, Attributed Idealized Influence; IM, Inspired Motivation; IS, Intellectual Stimulation; IC, Individualized Consideration; CR, Contingent Reward; MEP, Management-by-Exception Passive; LF, Laissez-Faire; IE-P, Emotional Intelligence Perception; IE-U, Emotional Intelligence Understanding; IE-R, Emotional Intelligence Regulation. Note2: R.W, Regression Weights; S.R.W., Standardized Regression Weights; E.E., Error estimation; C.R., Critical Ratio; EST, Estimations. Note3: *** Statistically significant relationship between variables at the level 0.005; ** Statistically significant relationship between variables at the level 0.01.

**Table 3:** Regression weights and standardized regression weights for university teaching.

Relationships between Variables			R.W.				S.R.W
			Estimate	E.E.	C.R.	*p*	EST
BII	←	TRANF-L	1.000	-	-	***	0.665
AII	←	TRANF-L	1.180	0.159	7.407	***	0.712
IM	←	TRANF-L	1.284	0.159	8.063	***	0.787
IS	←	TRANF-L	1.299	0.154	8.406	***	0.829
MEP	←	PAS-L	1.000	-	-	***	0.589
LF	←	PAS-L	0.916	0.267	3.436	***	0.494
IE-P	←	TRANF-L	3.222	2.432	1.325	0.185	0.725
IE-R	←	TRANF-L	−0.151	0.435	-0.346	0.729	−0.103
CR	←	TRANS-L	1.117	0.143	7.834	***	0.738
IC	←	TRANS-L	1.000	-	-	***	0.641
IE-P	←	PAS-L	−0.799	0.488	−1.637	0.102	−0.478
IE-R	←	TRANS-L	0.720	0.439	1.642	0.101	0.500
IE-U	←	TRANF-L	0.153	0.395	0.387	0.699	0.107
IE-P	←	TRANS-L	−3.261	2.326	−1.402	0.161	−0.761
IE-U	←	TRANS-L	0.602	0.406	1.484	0.138	0.427
IE-R	←	PAS-L	0.225	0.195	1.151	0.250	0.173
IE-U	←	PAS-L	0.372	0.202	1.845	*	0.292
TRANF-L	↔	PAS-L	−0.053	0.019	−2.839	**	−0.441
TRANS-L	↔	TRANF-L	0.115	0.021	5.406	***	0.997
TRANS-L	↔	PAS-L	−0.054	0.021	−2.608	**	−0.443

Note1: TRANF-L, Transformational Leadership; TRANS-L, Transactional Leadership; PAS-L, Passive Leadership; BII, Behavioral Idealized Influence; AII, Attributed Idealized Influence; IM, Inspired Motivation; IS, Intellectual Stimulation; IC, Individualized Consideration; CR, Contingent Reward; MEP, Management-by-Exception Passive; LF, Laissez-Faire; IE-P, Emotional Intelligence Perception; IE-U, Emotional Intelligence Understanding; IE-R, Emotional Intelligence Regulation. Note2: R.W, Regression Weights; S.R.W., Standardized Regression Weights; E.E., Error estimation; C.R., Critical Ratio; EST, Estimations. Note3: *** Statistically significant relationship between variables at the level 0.005; ** Statistically significant relationship between variables at the level 0.01; * Statistically significant relationship between variables at the level 0.05.
